# Study Protocol for a Prospective, Unicentric, Double-Blind, Randomized, and Placebo-Controlled Trial on the Efficacy of a Low-Histamine Diet and DAO Enzyme Supplementation in Patients with Histamine Intolerance

**DOI:** 10.3390/nu17010029

**Published:** 2024-12-25

**Authors:** Adriana Duelo, Sònia Sánchez-Pérez, Ana María Ruiz-Leon, Francesc Casanovas-Garriga, Salvador Pellicer-Roca, Irache Iduriaga-Platero, Judit Costa-Catala, M. Teresa Veciana-Nogués, Joaquim Fernández-Solà, Rosa M. Muñoz-Cano, Joan Bartra, Andrea Combalia, Oriol Comas-Basté, Rosa Casas, M. Luz Latorre-Moratalla, Ramon Estruch, M. Carmen Vidal-Carou

**Affiliations:** 1 Departament de Nutrició, Ciències de l’Alimentació i Gastronomia, Campus de l’Alimentació de Torribera, Universitat de Barcelona (UB), 08921 Santa Coloma de Gramenet, Spain; aduelo@ub.edu (A.D.); spellicer@ub.edu (S.P.-R.); iracheiduriaga98@ub.edu (I.I.-P.); jcostacatala@ub.edu (J.C.-C.); veciana@ub.edu (M.T.V.-N.); oriolcomas@ub.edu (O.C.-B.); mcvidal@ub.edu (M.C.V.-C.); 2 Institut de Recerca en Nutrició i Seguretat Alimentaria (INSA-UB), Universitat de Barcelona (UB), 08921 Santa Coloma de Gramenet, Spain; soniasanchezperez@ub.edu (S.S.-P.); amruiz@recerca.clinic.cat (A.M.R.-L.); frcasang7@alumnes.ub.edu (F.C.-G.); restruch@clinic.cat (R.E.); 3 Departament de Medicina Interna, Hospital Clínic de Barcelona, Universitat de Barcelona (UB), 08036 Barcelona, Spain; jfernand@clinic.cat; 4 Institut d’Investigacions Biomèdiques August Pi i Sunyer (IDIBAPS), 08036 Barcelona, Spain; rmunoz@clinic.cat (R.M.M.-C.); jbartra@clinic.cat (J.B.); 5 Centro de Investigación Biomédica en Red de Fisiopatología de la Obesidad y Nutrición (CIBEROBN), 28029 Madrid, Spain; 6 Fundación Dieta Mediterránea, 08021 Barcelona, Spain; 7 RICORS—Red Enfermedades Inflamatorias (REI), Insituto de Salud Carlos III, 28029 Madrid, Spain; 8 Servei d’Al lergologia, Hospital Clínic, Universitat de Barcelona (UB), 08036 Barcelona, Spain; 9 Departament de Dermatologia, Hospital Clínic de Barcelona, Universitat de Barcelona (UB), 08036 Barcelona, Spain; acombalia@clinic.cat

**Keywords:** histamine, histamine-related symptoms, DAO deficiency, intestinal microbiota, urinary histamine metabolites, histamine-restricted diet, food supplement

## Abstract

Background/Objectives: Histamine intolerance is primarily caused by a deficiency in the diamine oxidase (DAO) enzyme at the intestinal level. The reduced histamine degradation in the gut leads to its accumulation in plasma, thereby causing multiple clinical manifestations, such as urticaria, diarrhea, headache, dyspnea, or tachycardia, among others. The dietary management of this food intolerance consists of the follow-up of a low-histamine diet, often combined with DAO supplementation. To date, around twenty studies have investigated the effectiveness of these dietary strategies in reducing the frequency and/or intensity of symptoms, with promising results. However, the limitations of these studies (small patient cohort, lack of control group, and short dietary intervention periods) highlight the need for more ambitiously designed research. Therefore, the main objective of this prospective, unicentric, double-blind, randomized, and placebo-controlled trial is to evaluate the efficacy of a low-histamine diet and/or DAO supplementation over a three-month period in improving symptoms of histamine intolerance. Additionally, the impacts of these dietary strategies on the intestinal microbiota composition, urinary profile of histamine metabolites, serum DAO activity, and plasma histamine levels will be assessed throughout the intervention. Methods: The trial will enroll 400 patients who will be randomly assigned to one of two groups: the intervention group, which will follow a low-histamine diet, or the control group, which will maintain their habitual dietary habits. Within each of these groups, participants will be further divided into four subgroups to receive either exogenous DAO enzyme supplementation (from porcine or plant sources, with the latter administered at two different dosages) or a placebo. Therefore, a total of eight distinct intervention groups will be considered. The comparison of these groups will allow the evaluation of the individual effects of the low-histamine diet or DAO enzyme supplementation, as well as their possible synergistic effect. Results: The results of this study should help to improve dietary recommendations for histamine-intolerant patients and ultimately enhance their quality of life.

## 1. Introduction

**Figure 1:**
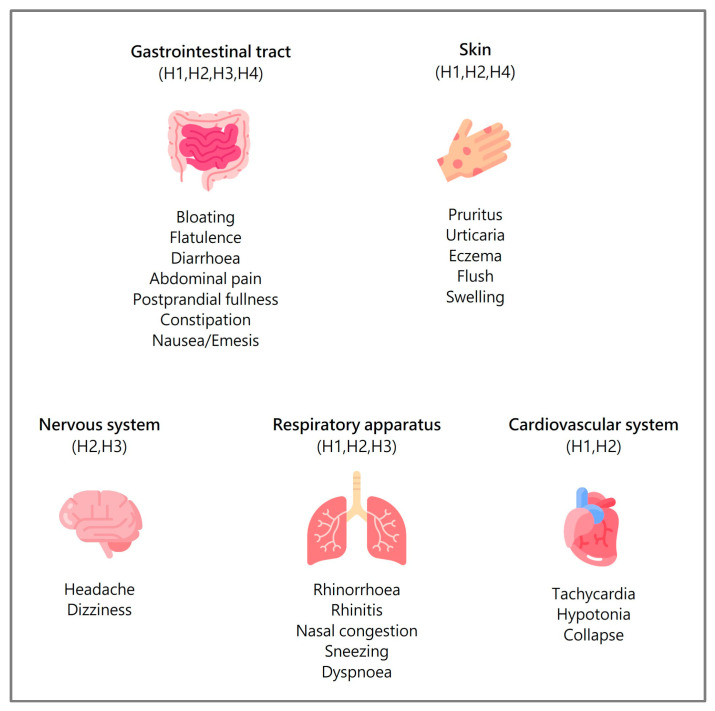
Main symptoms of histamine intolerance and the corresponding histamine receptors [1,4,9].

Histamine intolerance is a non-allergic hypersensitivity reaction that occurs in susceptible individuals when they ingest normally tolerable amounts of histamine. This condition arises primarily from deficient intestinal activity of the enzyme diamine oxidase (DAO) [1,2,3], which, by breaking it down, serves as the main barrier to dietary histamine entering systemic circulation. When DAO is deficient, histamine accumulates in the plasma, leading to clinical manifestations of histamine intolerance [4,5,6]. Histamine interacts with four types of histamine receptors in the body, with variable physiological effects. Consequently, symptoms resulting from histamine accumulation can impact multiple organs and tissues (Figure 1). Due to this wide range of effects, patients with histamine intolerance frequently exhibit complex combinations of symptoms [6,7,8,9].

The dietary management of histamine intolerance, aiming to reduce or avoid symptoms, is currently based on the exclusion of histamine-containing foods (i.e., a low-histamine diet), often combined with exogenous DAO supplementation to promote the metabolism of dietary histamine at the intestinal level [10]. There are two types of DAO supplements available on the market. The most common contains an active ingredient of porcine origin, whereas the recently introduced plant-based DAO supplements feature an active ingredient derived from lyophilized legume sprouts.

To date, around twenty studies have investigated the effectiveness of a low-histamine diet [11,12,13,14,15,16,17,18,19,20,21,22,23,24,25,26] or DAO supplementation [27,28,29,30,31] in reducing the frequency and/or intensity of histamine intolerance symptoms, with promising results. However, the limited number of clinical studies, many of them based on small patient groups or short dietary interventions, highlights the need for more ambitiously designed research to draw more accurate conclusions about the clinical effectiveness of these dietary strategies, applied separately or in combination.

Therefore, the main objective of this clinical study is to evaluate the efficacy of a low-histamine diet and/or DAO supplementation over a three-month period in improving symptoms of histamine intolerance caused by DAO deficiency. Additionally, the impacts of these dietary strategies on the composition of intestinal microbiota, urinary profile of histamine metabolites, serum DAO activity, and plasma histamine levels will be assessed throughout the intervention.

## 2. Materials and Methods

### 2.1. Study Design

This prospective, unicentric, double-blind, randomized, and placebo-controlled trial is being performed by a multidisciplinary team of researchers from the Food Bioactive Amines and Polyamines research group at the Food and Nutrition Campus, University of Barcelona, and the Cardiovascular Risk, Nutrition, and Aging research group at the August Pi i Sunyer Biomedical Research Institute (IDIBAPS) of the Hospital Clínic de Barcelona. The study commenced in June 2022 and is expected to conclude in May 2025.

**Figure 2:**
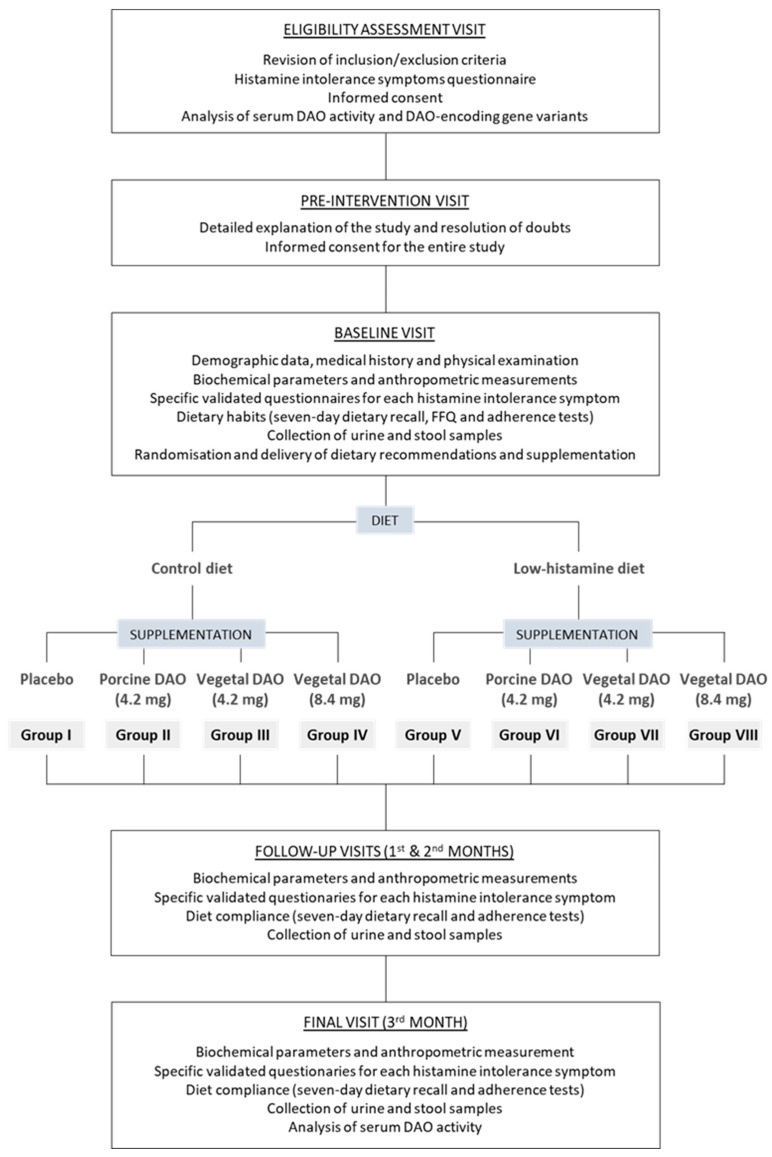
Schematic representation of the study design.

The study design is outlined in Figure 2. In this protocol, participants are randomized into eight study groups (50 individuals/group), based on combinations of diet (low-histamine or control diet) and supplementation (placebo, porcine DAO, and two different doses of plant-based DAO). The intervention period lasts three months.

The sample size is calculated considering the average number of symptoms reported by histamine-intolerant patients and the standard deviation (11.1 symptoms/patient ± 4.8) [6]. To detect a variation in at least two symptoms per patient, assuming an alpha risk of 0.05 and a beta risk of 0.02 in a bilateral contrast, a minimum of 42 participants per group is required. To accommodate a 10% drop-out rate, 50 participants will be recruited for each group. Therefore, as the study includes eight intervention groups, the total number of participants is 400.

#### 2.1.1. Recruitment and Eligibility Criteria

Participants are recruited during medical visits in the Allergology, Digestive, Internal Medicine, or Dermatology units of Hospital Clínic of Barcelona, based on the following eligibility criteria: age over 18 years, symptoms associated with histamine intolerance in two or more organs or systems, reduced serum DAO activity and/or any of the DAO-encoding gene variants (rs10156191, rs1049742, rs1049793, and s2052129), not having started a low-histamine diet, and not having taken DAO supplementation. The exclusion criteria are diagnosis of food or environmental allergies, pregnancy, and prescription of antibiotics and/or probiotics within the month prior to recruitment. To assess eligibility, potential participants undergo a questionnaire about histamine intolerance symptoms (Supplementary Figure S1) and an analysis of serum DAO activity and DAO-encoding gene variants, prior to signing an informed consent form (Figure 2). Serum DAO activity is analyzed using a radioimmunoassay technique, with a cut-off value of r < 12.54 U/mL indicating DAO deficiency. Genotyping of the DAO-encoding gene variants is performed by multiplex polymerase chain reaction (PCR) on the buffy coat fraction of blood samples, obtained after the centrifugation of blood collected in an EDTA vacutainer. Both analyses are conducted at the Echevarne Laboratory (Barcelona, Spain).

Eligible participants attend a pre-intervention visit, where the study objectives and procedures are explained in detail, and informed consent for the entire study is signed. Participants also receive a biological sample collection kit (for feces and urine) and instructions for proper collection procedures.

#### 2.1.2. Baseline Visit and Randomization of Participants

During the baseline visit, a trained nurse administers a questionnaire to record demographic data and a complete medical history, including alcohol consumption and smoking habits (Supplementary Figure S1). Additionally, participants complete various validated questionnaires specific to each symptom of histamine intolerance (described below). Comprehensive information on dietary habits is collected through a validated 151-item semi-quantitative food frequency questionnaire (FFQ), a seven-day dietary recall (for the seven days prior to the visit), and either a 14-item Mediterranean diet adherence screener (for control diet) or a low-histamine diet screener, administered by trained dieticians in face-to-face interviews [32].

The same trained personnel then collect anthropometric measurements, determine blood pressure (BP), and extract a blood sample to analyze biochemical parameters. Height is measured to the nearest 0.1 cm using a wall-mounted stadiometer, with subjects standing, wearing socks, and maintaining their heads in the horizontal plane. Body weight is recorded to the nearest 0.1 kg, with participants in light clothing, without shoes or accessories, and using a high-quality calibrated scale. Body mass index is calculated by dividing weight (kg) by the square of height (m^2^). Waist circumference is measured midway between the lowest rib and the iliac crest using an anthropometric tape, and hip circumference is measured at the maximum point below the waist, without compressing the skin [33]. BP is taken in triplicate after at least five minutes of rest using a validated semiautomatic oscillometer (Omron HEM-705CP, Hoofddorp, The Netherlands). The clinical analyses include a basic biochemical profile (i.e., glycemia, lipid profile, creatinine, urea, hepatic function parameters, and total proteins and albumin) and serum histamine and tryptase levels. All these analyses are performed in the CORE laboratory of Hospital Clinic following in-house routine procedures and meet all required quality criteria, receiving ISO 9001:2015 certification [34] and ISO 15189:2022 accreditation [35] for some tests.

At this point, participants provide urine and stool baseline samples for the study of metabolomic biomarkers of histamine and intestinal microbiota, respectively (described below). Samples are processed after collection and stored in −80 °C freezers.

Participants are randomized into eight groups, considering both diet and supplementation, as shown in Figure 2. Randomization is conducted using sealed envelopes, with participants selecting one envelope for the dietary intervention and another for the type of supplementation. Once an envelope is chosen, it is removed from the pool.

#### 2.1.3. Dietary Intervention

The low-histamine diet primarily involves the exclusion of foods likely to contain histamine (i.e., fish and its derivatives, fermented products, and certain plant-based foods), as well as foods with high amounts of other biogenic amines, such as citrus fruits, banana, nuts, pumpkin, and zucchini. Although these foods do not contain histamine, they may have significant amounts of putrescine and/or cadaverine, which could trigger the onset of histamine intolerance symptoms due to competition with histamine for degradation by the DAO enzyme [36]. Supplementary Figure S2 contains the dietary recommendations for participants, including a list of foods to consume and to avoid for correct adherence to the low-histamine diet. Participants in the control diet groups continue following their usual dietary habits.

Regarding supplementation, both possible origins of the DAO active ingredient (porcine and vegetal) will be assessed. In order to consider the potential influence of the dose of active ingredient, vegetal DAO supplements at two different doses will be tested. The posology for DAO (regardless of origin and dose) and the placebo involves taking one tablet 20 min before each main meal (three times/day). Porcine DAO supplementation consists of a kidney protein extract (4.2 mg), authorized as a novel food by the European Commission since 2017 [37]. Vegetal DAO supplements are formulated with lyophilized green pea (*Pisum sativum*) sprouts, containing either 4.2 mg or 8.4 mg of the active ingredient per tablet. The placebo tablets consist of microcrystalline cellulose, designed to match the form, size, and color of the DAO supplements. All tablets have an enteric coating to ensure their integrity during passage through the gastric environment. DR Healthcare España S.L.U. (Barcelona, Spain) provides both DAO supplements and placebo tablets.

#### 2.1.4. Follow-Up and Final Visits

The evolution of symptoms, urinary profile of histamine metabolites, and intestinal microbiota composition, as well as anthropometric measurements and blood biochemical parameters, including the basic biochemical profile and histamine and tryptase, are assessed after the first and second month (follow-up visits) and the third month (final visit) of the dietary intervention (Figure 2). Serum DAO activity will also be measured during the final visit.

Dietary compliance is assessed using seven-day food records and adherence tests for the low-histamine diet or Mediterranean diet [32]. Unlike the validated Mediterranean diet adherence screener, there is no standard test for assessing compliance with a low-histamine diet. The present research will validate the low-histamine diet adherence test prepared *ad hoc* for this study.

Throughout the intervention period, participants receive a phone call every two weeks to assess correct adherence to the study protocol and to monitor for any unexpected secondary effects.

### 2.2. Study Outcomes

#### 2.2.1. Symptoms of Histamine Intolerance

Symptoms related to histamine intolerance, listed in Figure 1, are evaluated through specific validated questionnaires as follows: (a) gastrointestinal manifestations are assessed by the Gastrointestinal Quality of Life Index (GIQLI) and Bristol Stool Scale [38,39]; (b) headaches by the Migraine Disability Assessment Scale (MIDAS) and Headache Impact Test (HIT-6) [40,41]; (c) respiratory symptoms by the Rhinoconjunctivitis Quality of Life Questionnaire (RQLQ) and Nasosinusual Symptoms Questionnaire (SNOT-22); and (d) dermatological manifestations by SCORing Atopic Dermatitis (SCORAD), the Itch Severity Scale (ISS), Quality of Life Index for Atopic Dermatitis (QoLIAD), and Dermatology Life Quality Index (DLQI) [42,43,44,45,46]. Additionally, the general quality of life of patients is assessed using the SF-36 Health Questionnaire [47].

Data obtained from these questionnaires will be used to evaluate the effectiveness of the dietary treatment in the improvement or remission of patient symptomatology. Differences among the study groups will be determined using covariance analysis with baseline parameters as covariates. A multivariate analysis adjusted for age, sex, and clinical parameters with significant baseline differences among groups will also be applied. These differences will be expressed as means (95% confidence intervals).

All collected data regarding symptomatology will also be used to assess potential correlations with other study outcomes, such the composition of intestinal microbiota, the urinary profile of histamine metabolites, serum DAO activity, plasma histamine levels, and DAO-encoding gene variants.

#### 2.2.2. Metabolic Biomarkers of Histamine in Urine

The urinary profile of histamine metabolites is assessed using both targeted and untargeted approaches to evaluate the potential impact of the dietary intervention. Spot urine samples (first morning urine) are collected and kept under refrigeration until delivery to the laboratory, where they are immediately aliquoted and stored at –80 °C until analysis. Prior to chromatographic analysis, urine samples are thawed, centrifuged, and diluted 50 µL 1:1 with Milli-Q water. Histamine and its three main metabolites (1-methylhistamine, imidazole acetic acid, and methylimidazole acetic acid) are identified and quantified by LC-ESI-MS/MS, using their respective deuterates as internal standards. Untargeted analysis is carried out by HPLC-TOF-MS according to the method described by Llorach et al. (2013) [48]. Data are extracted and aligned using commercial software, such as MarkerView 1.3 (Sciex, Framingham, MA, USA), or non-commercial software, such as MAIT [49]. Data analysis is conducted using unsupervised multiparametric tools, such as principal component analysis and hierarchical cluster analysis, as well as supervised multiparametric algorithms, such as PLS-DA with orthogonal signal correction [48,50].

#### 2.2.3. Intestinal Microbiota Composition

To study the profile and composition of the intestinal microbiota, bacterial DNA is isolated from stool samples using the QIAamp Powerfecal pro DNA kit (Qiagen GmbH, Hilden, Germany) according to the manufacturer’s instructions, and the DNA concentrations are determined by NanoDrop 2000 (Thermo Fisher Scientific, Wilmington, DE, USA). Subsequently, the V3–V4 region of bacterial 16S rRNA is sequenced on the Illumina MiSeq platform. Bioinformatic analysis for bacterial composition will be performed using the EzBioCloud Database (ChunLab, Inc., Seoul, Republic of Korea). Differences among study groups will be analyzed using the Kruskal–Wallis test. Alpha diversity will be described using the Shannon–Weaver Index and Simpson’s Index and beta diversity using Bray–Curtis and UniFrac methods [51].

### 2.3. Ethical Aspects and Confidentiality

The study is being carried out in accordance with the Declaration of Helsinki of 1975 and has been approved by the Bioethics and Clinical Research Committee of the Hospital Clinic de Barcelona (HCB/2022/0437). After approval, the study protocol was registered at Clinicaltrials.gov (ISRCTN64888465). All participants are informed of the study objectives and voluntarily sign an informed consent form.

All collected data are securely stored in a cloud-based spreadsheet with password-controlled access for researchers and a constant backup system. Privacy, anonymity, and confidentiality of all participant-identifying data are strictly maintained.

## 3. Discussion

The study described here is the first prospective, unicentric, double-blind, randomized, and placebo-controlled trial designed to evaluate the efficacy of combining a low-histamine diet with DAO supplementation in alleviating symptoms of histamine intolerance. Previous intervention studies have reported positive outcomes for either the low-histamine diet or DAO supplementation, although most of them have certain limitations regarding the number of patients, follow-up period, and control group [11,12,13,14,15,16,17,18,19,20,21,22,23,24,25,26,27,28,29,30,31]. In this sense, the broad majority of these studies (18 out of 21) considered small patient cohorts (fewer than 56 participants), 61% of which did not even exceed 30 patients. Although the studies conducted by Sienbenhaar et al. (2016) and Cucca et al. (2022) included larger sample sizes (157 and 146 patients, respectively), both trials entailed a significant drawback, due to the absence of a control group in their design [20,24]. In fact, the lack of a control group (i.e., not following a low-histamine diet or receiving placebo supplementation) is a recurring limitation in many of these studies (16 out of 21). Only the work performed by Izquierdo-Casas et al. (2019) included a cohort of 100 patients diagnosed with headache and DAO deficiency, with 50% receiving porcine DAO supplementation and the other half receiving a placebo [30]. Regarding the duration of the intervention, most of the studies, with the exception of three [13,18,22], were limited to a maximum of 4 weeks. For example, Mušič et al. (2013) considered a longer dietary intervention period of up to 12 months, during which patients with DAO deficiency followed a low-histamine diet. However, only 20 patients were monitored, and there was a lack of a control group [18]. The current study aims to address all these limitations by involving a total of 400 patients, including a placebo-controlled group and an intervention period of 3 months. The study design, with distinct intervention groups following a low-histamine diet and/or receiving DAO supplementation, will allow for the assessment of the impact of each treatment separately, as well as potential synergistic effects. Additionally, this clinical trial is pioneering in its evaluation of DAO sourced from plants as a dietary supplement for managing histamine intolerance.

Beyond symptom improvement, this study also examines the impact of dietary treatment on the intestinal microbiota composition in histamine-intolerant individuals. Although scientific literature on this topic is still limited, it has been hypothesized that intestinal dysbiosis could be a factor in the origin of this condition [51,52]. These previous studies coincide in describing a significant difference in bacterial diversity in those suffering from histamine intolerance, with an imbalance towards a higher presence of histamine-forming bacteria. Moreover, Sánchez-Pérez et al. (2022) identified a lower abundance of bacteria associated with gut health in patients diagnosed with histamine intolerance [51]. In another pilot study involving a small population, the same authors observed a reduction in the proportion of histamine-secreting bacteria and an increase in gut health-related species during the follow-up of a low-histamine diet [53]. By studying the intestinal microbiota composition, this study offers a foundation for future research to provide more scientific evidence about the role of gut microbiota in histamine intolerance, particularly whether intestinal dysbiosis is a cause or consequence of this food intolerance. These insights could guide subsequent investigations into the potential therapeutic role of probiotics in patients with dysbiosis-related histamine intolerance.

It is plausible to hypothesize that histamine intolerance due to DAO deficiency may lead to changes in the urinary metabolome, resulting in distinct metabolic phenotypes that can differentiate affected individuals. Indeed, in previous research, significant differences in the urinary concentration of methylhistamine were found, with markedly lower levels in individuals with histamine intolerance, compared to a control group [54]. In the current clinical trial, both targeted and untargeted metabolomic approaches in a large patient cohort will facilitate their validation as objective, reliable, and less-invasive biomarkers for histamine intolerance, providing the scientific basis for their future use as a complementary diagnostic tool alongside the current method, which primarily relies on patient anamnesis.

This study is also designed to provide valuable insights into the prevalence of genetic polymorphisms and levels of DAO activity and plasma histamine in patients with histamine intolerance. These parameters are correlated with the presence, intensity, and evolution of symptoms throughout the dietary intervention. In general, the results of this study should help to improve dietary recommendations for histamine-intolerant patients and ultimately enhance their quality of life.

The primary limitation of this study is that, despite the expected high number of participants (n = 400), the randomization into multiple intervention groups (low-histamine diet versus control, animal- and plant-based supplementation, and two dosage levels of the latter) results in a smaller sample size per group, which could potentially reduce the statistical power of the analyses. To address this, secondary analyses combining specific intervention groups, when methodologically appropriate, can be performed to effectively increase the sample size in those comparisons. Another limitation could be that the unicentric design means findings may not be generalizable to other histamine-intolerant populations and could be subject to selection bias among patients with this food intolerance. Finally, the reliability of dietary records may be influenced by recall bias or social desirability bias.

The main strength of the study is its design as a randomized clinical trial, which allows causal inference. Standardized data collection conditions for anthropometric and blood measurements, as well as for symptom recording and dietary intake frequency using validated questionnaires, are additional strengths. Overall, the findings of this clinical trial will contribute to the development of more effective and tailored dietary interventions for histamine intolerance. Specifically, robust and reliable evidence will be generated regarding the efficacy of the low-histamine diet, the current gold-standard treatment. Furthermore, the study will clarify the role of supplementation in symptom management, including the optimal type and dosage, thereby providing evidence-based recommendations for its use in clinical practice.

## Data Availability

The original contributions presented in the study are included in the article; further inquiries can be directed to the corresponding authors.

## Supplementary Materials

The following supporting information can be downloaded at: https://www.mdpi.com/article/10.3390/nu17010029/s1, Figure S1: Baseline questionnaires to obtain data on symptoms, demographic characteristics, and medical history; Figure S2: Excluded and allowed foods in the low-histamine diet.

## Abbreviations

DAO	Diamine Oxidase;
FFQ	Food Frequency Questionnaire;
PCR	Polymerase Chain Reaction;
EDTA	Ethylenediaminetetraacetic acid;
BP	Blood Pressure;
GIQLI	Gastrointestinal Quality of Life Index;
MIDAS	Migraine Disability Assessment Scale;
HIT-6	Headache Impact Test;
RQLQ	Rhinoconjunctivitis Quality of Life Questionnaire;
SNOT-22	Nasosinusual Symptoms Questionnaire;
SCORAD	SCORing Atopic Dermatitis;
ISS	Itch Severity Scale;
QoLIAD	Quality of Life Index for Atopic Dermatitis;
DLQI	Dermatology Life Quality Index;
LC-ESI-MS/MS	Liquid Chromatography-Electrospray Ionization-Tandem Mass Spectrometry;
HPLC-TOF-MS	High-Performance Liquid Chromatography-Time-of-Flight Mass Spectrometry;
MAIT	Metabolite Automatic Identification Toolkit
PLS-DA	Partial Least Squares Discriminant Analysis
