# Supplementary material for: Study Protocol for a Prospective, Unicentric, Double-Blind, Randomized, and Placebo-Controlled Trial on the Efficacy of a Low-Histamine Diet and DAO Enzyme Supplementation in Patients with Histamine Intolerance
Source: Nutrients. 2024 Dec 25;17(1):29. doi: 10.3390/nu17010029 (PMC11723128; doi:10.3390/nu17010029)
Supplement: Supplementary file 1 [file nutrients-17-00029-s001.zip › Figure S1.pdf]

Supplementary Figure S1. Baseline questionnaires to obtain data on symptoms, demographic characteristics, and medical history

General CRD - Nursing

Participant ID

____
(E.g.,DAO99)

Date

____

Sociodemographic Variables

Sex

○ Man ○ Woman ○ Other

Age

____

Place of Birth

○ **ANDALUSIA**
○ **ARAGON**
○ **ASTURIAS**
○ **CANTABRIA**
○ **CASTILLA Y LEÓN**
○ **CASTILLA-LA MANCHA**
○ **CATALONIA**
○ **CEUTA AND MELILLA**
○ **VALENCIAN COMMUNITY**
○ **EXTREMADURA**
○ **GALICIA**
○ **BALEARIC ISLANDS**
○ **CANARY ISLANDS**
○ **LA RIOJA**
○ **MADRID**
○ **MURCIA**
○ **NAVARRE**
○ **BASQUE**
○ **COUNTRY**
NOT IN SPAIN

NOT IN SPAIN (Specify the country)

____
(E.g., Morocco)

Marital Status

○ Single
○ Married
○ Widow/er
○ Divorced
○ Separated
○ Religious

What is the highest level of education you have completed?

○ Illiterate
○ Primary School
○ Secondary school or high
○ school Higher education
○ Insufficient data

Number of people you share your household with, excluding yourself:	______________________
Employment status:<br>Are you currently employed?	◯ Yes<br>◯ No<br>◯ On sick leave
¿What is your occupation?	______________________
¿Are you retired?	◯ Yes<br>◯ No
¿What was your occupation?	______________________

FAMILY HISTORY

Do you consume any type of alcoholic beverage?	◯ Yes<br>◯ No<br>◯ Former drinker
What types of alcohol do you consume?	______________________
Do you smoke tobacco?	◯ Yes<br>◯ No<br>◯ Former smoker
What type of tobacco do you smoke?	______________________
How much do you consume per day?	______________________

SYSTEMS / SYMPTOMS / DIAGNOSES

Gastrointestinal System: Do you have any of the following symptoms?

	Yes	No
Bloating	◯	◯
Flatulence	◯	◯
Post-meal fullness	◯	◯
Diarrhea	◯	◯
Abdominal pain	◯	◯
Constipation	◯	◯
Nausea	◯	◯
Vomiting	◯	◯
Heartburn	◯	◯

Do you have any symptoms affecting the gastrointestinal system?

◯ Yes
◯ No
(Select if you have any of the above)

Diagnoses: Gastrointestinal System

	Yes	No
Ulcerative colitis	◯	◯
Crohn's disease	◯	◯
Inflammatory bowel disease	◯	◯
Irritable bowel syndrome	◯	◯

Diagnoses: Gastrointestinal System - Other?

____

In the past month, have you taken any medication to treat gastrointestinal symptoms?

◯ Yes
◯ No

Medications: Gastrointestinal System

Generic name dose (mg/day)

Nervous System: Do you have any of the following symptoms?

	Yes	No
Headache	○	○
Dizziness	○	○

Do you have any symptoms affecting the nervous system?
○ Yes
○ No
(Select if you have any of the above)

Diagnoses: Nervous System

____

In the past month, have you taken any medication to treat symptoms related to the nervous system?
○ Yes
○ No

Medications: Nervous System

Generic name dose (mg/day)

Dermatological System

	Yes	No
Itching	○	○
Redness	○	○
Hives	○	○
Eczema – Atopic dermatitis	○	○
Sweating	○	○

Do you have any symptoms affecting the dermatological system?
○ Yes
○ No
(Select if you have any of the above)

Diagnoses: Dermatological System

____

In the past month, have you taken any medication to treat symptoms related to the dermatological system?
○ Yes
○ No

Medications: Dermatological System

Generic name dose (mg/day)

Cardiovascular System: Do you have any of the following symptoms?

	Yes	No
Tachycardia	◯	◯
Hypotonia	◯	◯
Fainting/collapses	◯	◯

Do you have any symptoms affecting the cardiovascular system?

◯ Yes
◯ No
(Select if you have any of the above)

Diagnoses: Cardiovascular System

____

In the past month, have you taken any medication to treat symptoms related to the cardiovascular system?

◯ Yes
◯ No

	Yes	No
Medications for dyslipidemia	◯	◯
Medications for blood pressure	◯	◯
Aspirin, Adiro or similar	◯	◯

Medications: Cardiovascular System

Generic name dose (mg/day)

Respiratory System: Do you have any of the following symptoms?

	Yes	No
Rhinorrhea	◯	◯
Rhinitis	◯	◯
Nasal Congestion	◯	◯
Sneezing	◯	◯
Dyspnea	◯	◯

Do you have any symptoms affecting the respiratory system?

◯ Yes
◯ No
(Select if you have any of the above)

Diagnoses: Respiratory System

	Yes	No
Sleep apnea	◯	◯
Chronic bronchitis-Emphysema	◯	◯

Diagnoses: Respiratory System - Other?

____

In the past month, have you taken any medication to treat symptoms related to the respiratory system?

◯ Yes
◯ No

Medications: Respiratory System

Generic name dose (mg/day)

Do you have any other symptoms?

____

Do you have any other affected systems?

____

Do you have any of the following diagnoses?

	Yes	No
Fibromyalgia	◯	◯
Chronic fatigue	◯	◯
Type 2 diabetes	◯	◯
Multiple chemical sensitivity	◯	◯
Anxiety	◯	◯
Depression	◯	◯
Anemia	◯	◯
Food intolerance	◯	◯
Dysautonomia	◯	◯
Severe allergy	◯	◯
Cancer or tumors	◯	◯

Diagnoses: Other?

____

In the past month, have you taken any other medication, supplement or complementary product?

	Yes	No
Antihistamines	○	○
Antibiotics	○	○
Probiotics	○	○
Vitamins or minerals	○	○
Corticosteroids	○	○
Other pain or fever relievers	○	○
Tranquilizers, sedatives or	○	○
anxiety medication	○	○
Hormonal treatment (women)	○	○
Insulin	○	○
Oral antidiabetic medication	○	○
Supplementation with DAO enzyme	○	○

Other:
Generic name dose (mg/day)

Have you already started a low-histamine diet?	○	○

Physical Examination

PAS 1	____________________
PAS 2	____________________
PAS 3	____________________
PAS mean	____________________
PAD 1	____________________
PAD 2	____________________
PAD 3	____________________
PAS mean	____________________
FC 1	____________________
FC 2	____________________
FC 3	____________________
FC mean	____________________

General CRD – Group - Dietitian

Participant ID	______________________ (e.g. DAO99)
Date of visit	______________________

ANTHROPOMETRY

Weight (kg)<br>(Use "." as the separator, e.g. 58)	______________________
Size (m)<br>(Use "." as the separator, e.g. 1.58)	______________________
IMC (kg/m^2)	______________________
Waist (cm)<br>(Use "." as the separator, e.g. 78.5)	______________________
Hip (cm)<br>(Use "." as the separator, e.g. 95.5)	______________________
