# Supplementary material for: Study Protocol for a Prospective, Unicentric, Double-Blind, Randomized, and Placebo-Controlled Trial on the Efficacy of a Low-Histamine Diet and DAO Enzyme Supplementation in Patients with Histamine Intolerance
Source: Nutrients. 2024 Dec 25;17(1):29. doi: 10.3390/nu17010029 (PMC11723128; doi:10.3390/nu17010029)
Supplement: Supplementary file 1 [file nutrients-17-00029-s001.zip › Figure S2.pdf]

Supplementary Figure S2. Excluded and allowed foods in the low-histamine diet

FOOD CATEGORY	EXCLUDED FOODS	ALLOWED FOODS
CEREALS AND TUBERS	-	Rice, oats, spelt, corn, millet, quinoa, wheat, buckwheat, sweet potato, potato, cassava, etc.
FRUITS AND NUTS	Strawberries and red berries, papaya, avocado, citrus fruits (orange, grapefruit, mandarin, lemon, kiwi and pineapple), banana, plums, nuts (walnut, peanut, almond, cashews and hazelnut)	Apricot, cherries, pomegranate, fig, mango, apple, peach, melon, pear, grape, watermelon, pine nuts, etc.
VEGETABLES AND GREENS	Vegetable pickles (e.g., sauerkraut, olives), zucchini, pumpkin, spinach, eggplant and tomato	Chard, artichoke, broccoli, onion, fennel, green beans, asparagus, lettuce, bell pepper, beet, carrot, etc.
DAIRY PRODUCTS AND SUBSTITUTES	Aged, semi-aged, and grated cheeses, milk and yogurt	Fresh cheeses, plant-based drinks, fermented products made from soy or other plants (plant-based "yogurt")
LEGUMES AND DERIVATIVES	Sufu and tempeh	Beans, chickpeas, lentils, soybeans, etc.
FISH AND SEAFOOD	Canned or semi-canned fish (canned tuna, anchovies, sardines or mackerel, anchovies in vinegar, smoked salmon, etc.) and seafood	Fresh or frozen white or blue fish*, cuttlefish, squid, octopus, etc.
EGG	Egg white	Yolk
MEAT AND POULTRY	Sausages (*fuet, chorizo, longaniza*, etc.), viscera (liver, foie, kidney, etc.)	Fresh or frozen meat*, cured ham or loin, cooked ham or turkey, mortadella, Frankfurt, etc.
DRINKS	Orange juice, tomato juice, alcoholic beverages (cava, wine, beer, cider, spirits) and tea	Water, infusions, coffee, etc.
FATS	-	Olive oil and seeds, toasted sesame cream (tahin), butter and margarine
CONDIMENTS	Vinegar, soy sauce and tomato sauce	Oregano, basil, turmeric, ginger, mint, salt, sugar, honey and sweeteners
OTHERS	Chocolate, citrus jam, pastries and cakes containing excluded foods	Carob, compotes and sorbets made from suitable fruits, chia and flax seeds, sunflower and pumpkin seeds

*It is important to ensure that both fish and meat are very fresh at the time of purchase. Otherwise, it is preferable to buy them directly frozen.

Reference; Sanchez-Perez, S., Comas-Basté, O., Veciana-Nogués, MT., Latorre-Moratalla, ML., Vidal-Carou, MC. 2021. Low-histamine diets: Is the exclusión of Foods justified by their histamine content?.*Nutrients*, 13, 1395.
